# Knowledge, Attitude, and Practice of Cigarette Smoking Among Medical Students of Quaid-E-Azam Medical College, Bahawalpur: A Web-Based Cross-Sectional Study

**DOI:** 10.7759/cureus.46459

**Published:** 2023-10-04

**Authors:** Nawal Mustafa, Ayesha Bashir, Rohab Sohail, Satesh Kumar, Mahima Khatri, Giustino Varrassi

**Affiliations:** 1 Medicine, Quaid-e-Azam Medical College, Bahawalpur, PAK; 2 Internal Medicine, Quaid-e-Azam Medical College, Bahawalpur, PAK; 3 Medicine and Surgery, Shaheed Mohtarma Benazir Bhutto Medical College, Karachi, PAK; 4 Medicine and Surgery, Dow University of Health Sciences, Karachi, PAK; 5 Pain Medicine, Paolo Procacci Foundation, Rome, ITA

**Keywords:** cigarette, college, smoking, medical, students

## Abstract

Cigarette smoking acknowledged as the foremost contributor to preventable illnesses and deaths, has steadily risen since its inception, evolving into a global health crisis of paramount significance, particularly within the context of medical students who represent the future healthcare workforce. This study, conducted at Quaid-e-Azam Medical College, Bahawalpur, aimed to comprehensively evaluate current students' knowledge, attitudes, and practices concerning cigarette smoking. Employing a web-based cross-sectional observational descriptive study design over the study period from June 15, 2020, to August 1, 2020, a sample of 200 medical students drawn from the first to fifth year was examined using stratified sampling. Data collection involved the distribution of a meticulously designed and pre-tested questionnaire through social media platforms, encompassing inquiries about participants' biodata and research-related topics. The subsequent data analysis utilized Google Spreadsheets, Microsoft Excel, Microsoft Word, and SPSS software to calculate percentages, create graphical representations, construct tables, and apply the chi-square test. The survey findings illuminated a remarkably high level of awareness, with 99% of respondents recognizing the harmful effects of smoking, including elevated cancer risk, and 94% acknowledging its addictive nature. A substantial 93% regarded passive smoking as equally detrimental as active smoking. In comparison, 98.5% were aware of the heightened risk of respiratory illnesses in children exposed to smoking. The majority exhibited a responsible stance towards discouraging smoking, with 90.5% feeling a duty to encourage cessation and 71.5% considering maintaining good health a sufficient incentive to quit. Additionally, 97% concurred that smoking in the presence of children should be avoided. Concerning smoking cessation, 64.5% believed high taxes were effective, and 97.5% deemed public smoking bans effective measures. Notably, 74% thought professional advice had limited influence on a smoker's attitude. Active smokers constituted a mere 20% of the sample, with a mere 2% commencing smoking before age 16 and 10% succumbing to peer pressure or media influence as instigating factors. Furthermore, 13.5% reported exam-related anxiety as a trigger for smoking, and 10% admitted to smoking in the company of non-smokers. Encouragingly, 19.5% expressed a desire to quit, with 5% seeking professional guidance in their cessation attempts. In conclusion, most participants exhibited commendable knowledge and a positive attitude towards cigarette smoking, contributing to a low prevalence of tobacco consumption among them. Nevertheless, the study underscores the need for ongoing improvements through targeted educational initiatives and governmental regulations to further mitigate tobacco use among future healthcare professionals and the broader population.

## Introduction

Cigarette smoking is described as the act of inhalation and exhalation of the fumes produced by burning tobacco [1], usually rolled in thin paper. Since the origin of cigarettes in the 20th century, their worldwide manufacture and utilization have been on a constant rise [2], making cigarette smoking a global epidemic [3]. It is primarily concentrated in developing countries [4], like Pakistan, where illiteracy and ignorance about its harmful effects [5] have made it a national catastrophe, with approximately 1.3% female and 27.6% male smokers [6]. The male smokers in Pakistan alone amount to the majority (45%) of the total smokers in South Asia [5].

The World Health Organization (WHO) states that cigarette smoking takes more than 3.5 million lives each year, and this figure is expected to rise to 10 million per year by 2030 [3], making cigarette smoking the principal cause of avoidable morbidities and mortalities [7]. Out of the 40,000 active agents in cigarette smoke, over 40 are cancerous [8], including tar and heavy metals, while others like nicotine and carbon monoxide are detrimental to several organ systems of the body. Smoking is associated with several diseases [4,9], including chronic obstructive pulmonary disease, cerebrovascular diseases like stroke and cataracts, cardiovascular diseases like coronary artery disease, and carcinomas of the oral cavity, lung, pancreas, bladder, and kidneys [3,5,7]. It is also injurious to the health of pregnant women and their babies, even if taken passively, causing stillbirth, low birth weight, miscarriage, and congenital anomalies [5,10]. The financial implications due to the premature disease and death of the working force lead to a decline in the growth of low- and middle-income countries, where the economy is already facing a downward spiral [11]. The adverse effects of smoking are not only limited to smokers but also extend to non-smokers due to the inhalation of secondhand smoke, which has even greater numbers of carcinogenic constituents than first-hand cigarette smoke and, hence, is even more hazardous. Acute respiratory diseases are 50-100% more likely to develop in children exposed to passive smoking than non-exposed children. Studies indicate that around one-third of adults worldwide have frequent exposure to secondhand smoke [7].

A variety of distinct trends in smoking have been observed among medical students all around the world. In Pakistan, over half of the smoker medical students (51.7%) began smoking after scoring admission to medical college [1]. Multiple factors contribute to the initiation and adoption of this practice, some of the primary being peer pressure, hostel life, social acceptance, life troubles, alleviation of anxiety, stress, anger, and frustration, smoking prevalence in the family, illiteracy of parents, advertisement through media, e.g., films, and the craving caused by nicotine contained in cigarettes [1,3,12]. Thus, despite having a keen insight into the health hazards of both active and passive smoking, medical students smoke in multitudes, leading to a consumption rate equal to or even higher than that of their non-medical colleagues [13,14]. Being a respected and privileged part of society, healthcare professionals are not only heard but also heeded. However, the general population also views them as role models [5,12]. Thus, smoking among medical students has ramifications that can only be addressed through education and counseling [15]. Although the smoking rate among medical professionals, including medical students, has decreased in several developed countries, it remains inconsistent in developing countries [12].

In Pakistan, regulation of tobacco consumption has aptly been proclaimed as one of the essential pillars of the National Action Plan for Control of Non-Communicable Diseases (NAP-CND) [16]. Major preventive approaches include prohibiting smoking advertisements via media, national awareness campaigns regarding the dangers of smoking [16], and placing mandatory warnings on cigarette packets covering about 33% of the front and back [17]. Medical students' knowledge, attitudes, and practices related to smoking prevention and cessation can be enhanced through appropriate education and targeted training during their medical years [18]. Management of tobacco addiction has been suggested to aid in smoking cessation as tobacco-related morbidities reduce and the overall standard of living improves with quitting [19]. Despite relentless attempts by all public and private organizations to control cigarette smoking in Pakistan, it has been on the rise [2]. This study was conducted to explore the association between medical life and smoking through the assessment of its knowledge, attitude, and practice among the students of Quaid-e-Azam Medical College. The study's findings aim to provide relevant data to support targeted interventions to reduce the prevalence of smoking among medical students in the future.

## Materials and methods

In the pursuit of a comprehensive understanding of the subject matter, a meticulously designed methodology was employed for this research study. The study setting was among the current students of Quaid-e-Azam Medical College, Bahawalpur, providing a relevant and representative sample. To capture the multifaceted dimensions of the research objectives, a web-based cross-sectional observational descriptive study design was implemented, conducted over a specific duration from June 15, 2020, to August 1, 2020. The sampling population comprised current medical students spanning from the first year to the fifth year of their medical education at Quaid-e-Azam Medical College, Pakistan, ensuring a diverse representation of the student body. A judiciously selected sample size of 200 students was meticulously chosen through the application of a stratified sampling technique, ensuring that the demographics and characteristics of the participants were appropriately considered. Ethical considerations played a pivotal role in the research process. The inclusion criteria encompassed willing students of both genders affiliated with Quaid-e-Azam Medical College who actively consented to participate in the study. In contrast, the exclusion criteria thoughtfully excluded students who were unwilling to take part or belonged to different medical institutions, ensuring the integrity of the study's scope.

The ethical framework governing this research was underpinned by the procurement of online informed consent from all participants before data collection, a fundamental step in respecting the autonomy and rights of the individuals involved. The participants were also explicitly informed of their right to withdraw from the study at any point, underscoring the importance of voluntariness and informed decision-making. Furthermore, stringent measures were adopted to safeguard the confidentiality of the collected data, which was securely stored on password-protected computers to ensure data privacy and anonymity. The data collection process was executed with precision, employing a pre-designed and pre-tested questionnaire administered through Google Forms. The questionnaire was thoughtfully structured into two distinct sections: the first section was dedicated to collecting participants' biodata, and in contrast, the second delved into questions about the research topic. The questionnaire was disseminated via social media platforms with a user-friendly link. To ensure the integrity of the data collected, online informed consent was meticulously acquired from each participant, and eligibility criteria were rigorously verified before data entry.

The process of data analysis was marked by a systematic approach, wherein questions were methodically grouped based on their relevance to knowledge, attitude, or practice. The data obtained from participant responses were subjected to rigorous analysis, leveraging tools such as Google Spreadsheet, Microsoft Excel, and Microsoft Word. These analytical tools facilitated the calculation of percentages and the generation of graphical representations and tables, enhancing the presentation of the research findings. Furthermore, to assess the significance of the collected data, the chi-square test, a robust statistical tool, was expertly applied through the Statistical Package for the Social Sciences (SPSS) software.

In summary, the material and methods employed in this research were characterized by a meticulous and ethical approach, ensuring the robustness and integrity of the study. This comprehensive methodology served as the cornerstone for the acquisition of valuable insights into the knowledge, attitudes, and practices of medical students regarding cigarette smoking.

## Results

Demographic profile

This study was conducted on a sample of 200 medical students of Quaid-e-Azam Medical College, Bahawalpur, with an equal number of male and female participants, i.e., 100 each, with an average male age of 21.40 and an average female age of 20.94. Equal representation of each class from first to fifth year was ensured, i.e., 20% (40 students from each class), of which 68.5% were hostilities and 31.5% were day scholars.

Knowledge

The study revealed that almost all participants, i.e., 99% (males=100%, females=98%), were familiar with the harmful effects of smoking; an equal percentage of 99% (males=98%, females=100%) were aware that cigarette smokers are more susceptible than non-smokers to certain cancers like oral, laryngeal, lung, etc. Out of these, 94% (males=88%, females=100%) also knew that the tobacco contained in cigarettes can lead to addiction. Upon inquiry regarding passive smoking, 93% (males=91%, females=95%) had an idea that it is as dangerous as active smoking, and 98.5% (males=97%, females=100%) knew that it increases the risk of respiratory illnesses among children. In addition, 79.5% (males=72%, females=87%) were aware that occasional smoking is also injurious to health. As far as smoking cessation is concerned, 64.5% (males=67%, females=62%) believed e-cigarettes to be of no use in quitting, and 84% (males=83%, females=85%) thought that cessation even in chronic smokers suffering from irrevocable health damage is beneficial. More than half of the respondents, i.e., 68.5% (males=74%, females=63%), were acquainted with the current guidelines for the management of cigarette smoking, given in Table 1.

**Table 1:** Knowledge of respondents about cigarette smoking

Statement	Males (n=100) (n=%)		Females (n=100) (n=%)		Pearson Chi-Square	P. Value
	Yes (%)	No (%)	Yes (%)	No (%)		
Knowledge about harmful effects of smoking	100	0	98	2	2.020	0.155
Cigarette smoking can lead to addiction	88	12	100	0	12.766	0.000
Passive smoking is as dangerous for health as active smoking	91	9	95	5	1.229	0.268
Passive smoking among children increases their risk of respiratory illnesses	97	3	100	0	3.046	0.081
Occasional smoking is not injurious to health	28	72	13	87	6.903	0.009
E-cigarettes are useful in quitting smoking	33	67	38	62	0.546	0.460
Smokers are more susceptible to certain cancers i.e. oral, laryngeal, lung etc.	98	2	100	0	2.020	0.155
Smoking cessation in chronic smokers is not beneficial because of irrevocable health damage	17	83	15	85	0.149	0.700
Acquainted with the current guidelines for management of smoking	74	26	63	37	2.804	0.094

Attitude

In Table 2, the attitude of participants towards smoking was assessed. Results revealed that only 16% (males=21%, females=11%) viewed cigarette smoking as fashion. Instead, being medical students, 90.5% (males=84%, females=97%) considered it their responsibility to encourage others to quit, out of which 71.5% (males=66%, females=77%) thought that maintenance of good health was a sufficient reason for smoking cessation. Of the participants, 97% (males=96%, females=98%) also agreed that adults should avoid smoking in the company of children. Regarding smoking regulation, more than half of the participants, i.e., 64.5% (males=53%, females=76%), believed that high taxes on cigarette packets would be effective, and the majority, i.e., 97.5% (males=95%, females=100%) agreed that smoking should be prohibited at public places. According to 74% of respondents (males=75%, females=73%), professional advice regarding smoking cessation has little impact on smokers' attitudes.

**Table 2:** Attitude of respondents towards cigarette smoking

Statement	Males (n=100) (n=%)		Females (n=100) (n=%)		Pearson Chi-Square	P. Value
	Yes (%)	No (%)	Yes (%)	No (%)		
Knowledge about harmful effects of smoking	100	0	98	2	2.020	0.155
Cigarette smoking can lead to addiction	88	12	100	0	12.766	0.000
Passive smoking is as dangerous for health as active smoking	91	9	95	5	1.229	0.268
Passive smoking among children increases their risk of respiratory illnesses	97	3	100	0	3.046	0.081
Occasional smoking is not injurious to health	28	72	13	87	6.903	0.009
E-cigarettes are useful in quitting smoking	33	67	38	62	0.546	0.460
Smokers are more susceptible to certain cancers i.e. oral, laryngeal, lung etc.	98	2	100	0	2.020	0.155
Smoking cessation in chronic smokers is not beneficial because of irrevocable health damage	17	83	15	85	0.149	0.700
Acquainted with the current guidelines for management of smoking	74	26	63	37	2.804	0.094

Practice

**Figure 1:**
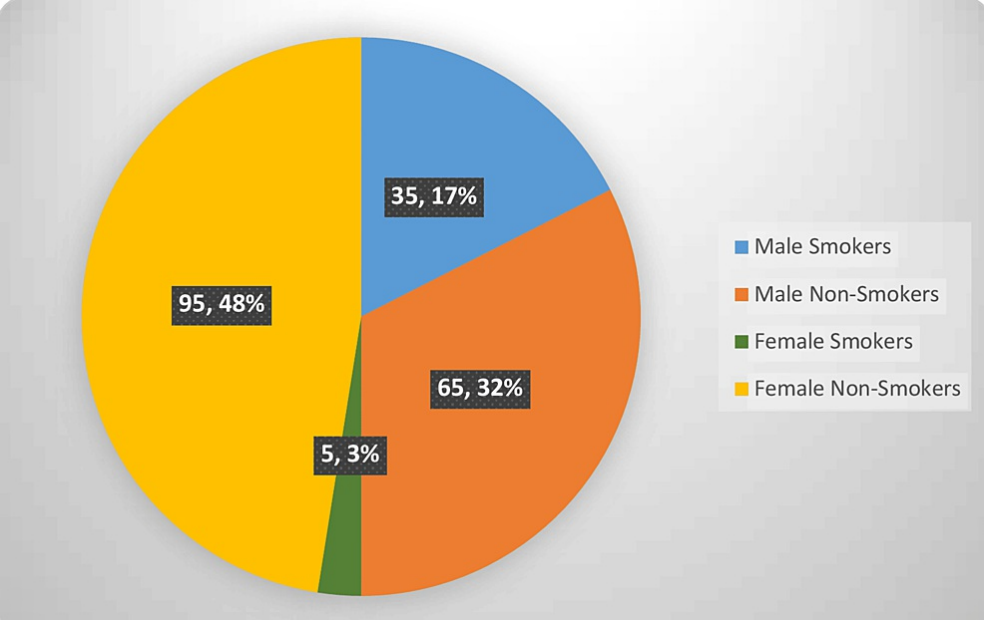
Prevalence of smoking among male and female medical student Pearson Chi-Square= 28.125, P. Value= 0.000

**Figure 2:**
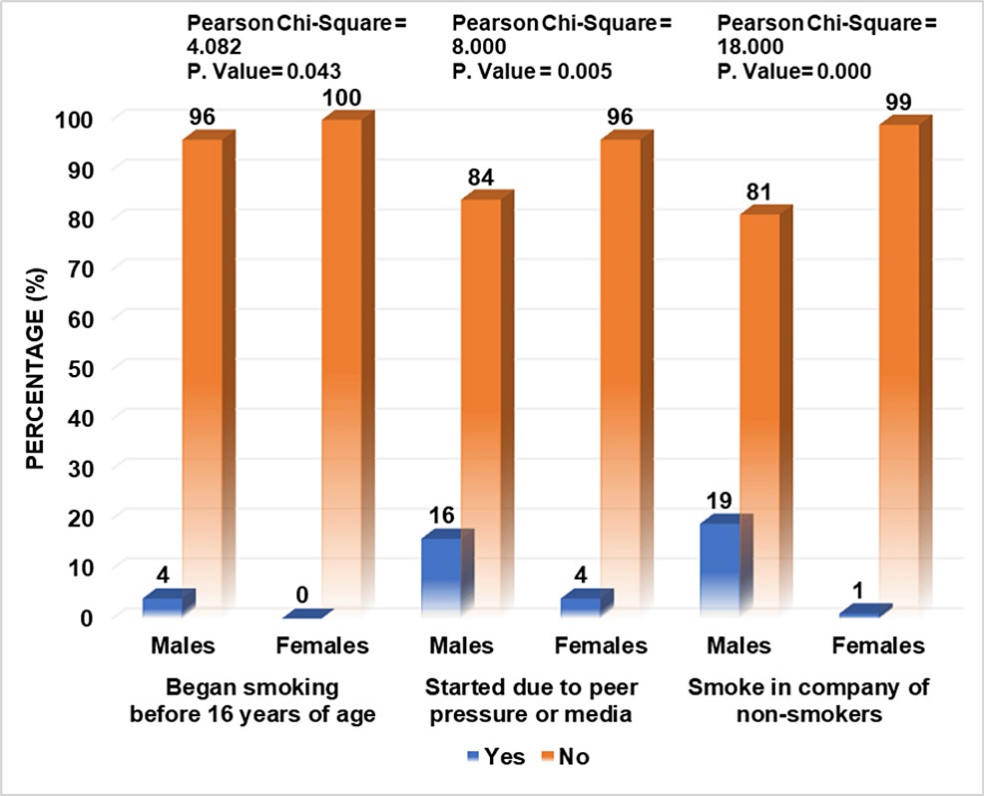
Practices of respondents regarding cigarette smoking

**Figure 3:**
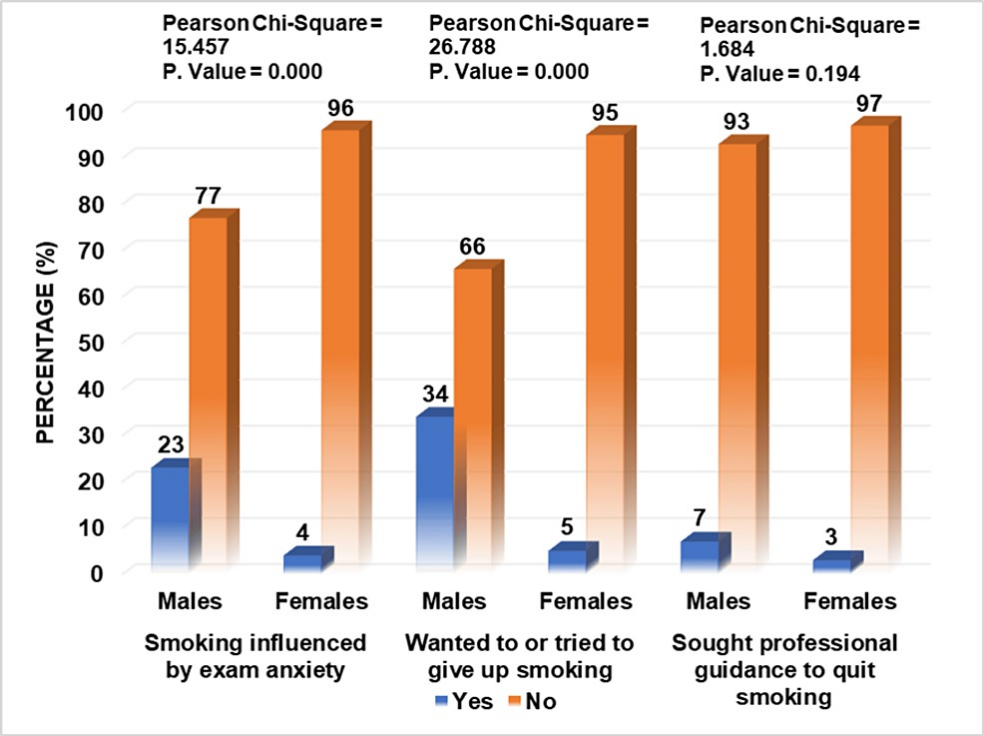
Practices of respondents regarding cigarette smoking

Further inquiry revealed that only a small percentage of respondents, i.e., 20% (males=35%, females=5%) were active cigarette smokers, out of which only 2% (males=4%, females=0%) started smoking before the age of 16, with 10% (males=16%, females=4%) declaring peer pressure or media as their primary reason for taking up smoking. The anxiety of professional examinations was found to influence the smoking habit of 13.5% (males=23%, females=4%) of participants, and 10% (males=19%, females=1%) also admitted to practicing smoking in the company of non-smokers. Regarding smoking cessation, 19.5% (males=34%, females=5%) wanted or tried to give up smoking in the past, out of which 5% (males=7%, females=3%) even sought professional guidance for it, as given in Figure 1, 2, 3.

## Discussion

This study assessed the knowledge, attitude, and practice of cigarette smoking among Quaid-e-Azam Medical College medical students in Bahawalpur. A sample of 200 students was interviewed online; the results were compiled and compared with research carried out in Pakistan, India, Bangladesh, Saudi Arabia, Argentina, Canada, Kosovo, and Mongolia [1-8].

Knowledge

As the study was conducted on medical students, adequate knowledge regarding the hazards of smoking was expected to be present due to their higher level of education, which was in accordance with the result obtained, according to which 99% of all respondents were familiar with the harmful effects of smoking. This was similar to a study conducted in Jeddah, Saudi Arabia, where 94% of participants had this knowledge [13]. This conclusion was further reinforced when queried regarding smoking-associated morbidities, as the majority, i.e., 99%, knew it increased susceptibility to certain cancers such as oral, laryngeal, lung, etc. This result is almost equal to that obtained in research in West Bengal, India, where 100% and 97% of participants believed it increased the risk for lung and oral/laryngeal cancer, respectively [7]. Nicotine addiction and dependence are well-known phenomena. 94% of respondents in this study and a lesser percentage, i.e., 65.9% of males and 77.3% of females, in a study conducted in Najran, Saudi Arabia, believed that cigarette smoking could lead to addiction [10].

Passive smoking is the inhalation of cigarette smoke by people other than active smokers and is known to cause significant disease, disability, and death. Of the students, 93% thought it to be as harmful as active smoking, which is in contrast to a study conducted in Prishtina, Kosovo, where only 38.50% were of this view, while 38.80% believed it to be more harmful and 22.60% believed it to be less harmful than active smoking [15]. Children frequently exposed to second-hand smoke also have an increased risk of contracting respiratory illnesses like flu, asthma, etc. In this study, 98.5% of respondents and 77% of research carried out in Jeddah, Saudi Arabia, had this knowledge [13]. Despite adequate knowledge, 20.5% of students believed that occasional smoking was not injurious to their health. The rationale behind this might be their justification for smoking on a non-daily basis for fun at parties and friend gatherings. In a study performed in Argentina, only 6% held this opinion [12].

E-cigarettes have recently become popular as healthier alternatives to tobacco smoking with the potential to aid in quitting; however, 64.5% of participants considered them to be of no help in smoking cessation. This contrasts with research conducted in Karachi, Pakistan, where only 28.3% were of this view [6]. Although the health risks of smoking increase with the duration of smoking, quitting at any age is still beneficial. This was supported by 84% of participants who agreed that quitting even for chronic smokers is helpful, following an analysis in Argentina where 98.5% were of this notion [12]. More than half of the participants, i.e., 68.5%, were acquainted with the current guidelines for the management of cigarette smoking. In contrast, in research conducted in Canada, less than 10% of respondents in any year and about 30% in the final year had this knowledge [18].

Attitude

Recently, cigarette smoking has been viewed as a form of fashion by the general population due to its blatant portrayal by the media through films and movies. However, detailed analysis revealed a minimal correlation between style and smoking habits among medical students, as only 16% of students in this study and a somewhat higher percentage, i.e., 48% in a survey designed in Taif, Saudi Arabia, considered smoking as fashion or a good habit [3]. Instead, being medical students, 90.5% of respondents in this study and 85.1% in research done in Argentina considered it their responsibility to encourage others to quit smoking [12]. The rationale behind this attitude might be their higher level of education and increased awareness regarding the hazards of smoking. According to 71.5% of participants, maintaining good health is a sufficient reason to quit smoking, almost equal to the response received in an analysis in Taif, Saudi Arabia, where 75.59% thought so. Perhaps the reason is their awareness that quitting improves the general quality of life and increases the average life span of smokers by reducing the risk of life-threatening complications [3]. Also, 97% agreed that adults should avoid smoking in the company of children; this result is similar to the 96% obtained in a study performed in Jeddah, Saudi Arabia [13]. This view not only reflects upon the health risks of second-hand smoke in children but also highlights the normalization of smoking practice for them, which inevitably leads to their adoption of this habit later in life.

Regarding smoking regulation, more than half of the students, 64.5%, agreed that high taxes on cigarette packets would be effective, which is in conjunction with research conducted in West Bengal, India, where 70% thought so [7]. Students are financially dependent on their families and have limited budgets, so any legislation regarding the increase in the price of cigarettes would lead to a fall in their consumption. Regarding implementing the government policy to ban cigarette smoking in public places, 97.5% of respondents in this study and 90% in a study performed in West Bengal, India, gave an encouraging response. Despite strong agreement, the government fails to take appropriate action [7]. Regarding smoking cessation, 74% of participants believed that professional advice has little impact on a smoker's attitude, while research done in Argentina showed contrasting results, where only 30.9% believed so [12]. The difference found in the results is likely due to the lower literacy rate and awareness in the general population of Pakistan compared to Argentina, leading to a higher level of ignorance towards doctors' advice.

Practices

Cigarette smoking has rapidly become a global health problem. Upon inquiry regarding smoking practices, 20% of respondents were found to be active cigarette smokers, almost equal to the 19.76% of active smokers found in the study in Muzaffarnagar, India [2]. The reason behind this relatively low prevalence in medical students compared to the general population is their higher education level and orientation towards becoming future doctors. Among medical students, the smoking rate, especially low in females, is attributed to their lower socioeconomic status and social disapproval of women who smoke. The prevalence of smoking among adolescents generally increases with age; this is in accordance with the results found, as only 2% of participants in this study began smoking before age 16. This is slightly less than the result obtained in research conducted in Prishtina, Kosovo, i.e., 12.70% [15].

When asked about reasons for smoking, 10% stated peer pressure or the media as a significant factor. In contrast, according to a study in Gujrat, India, 85.7% attributed it to peer pressure, and 4.7% claimed it to be due to the media. This indicates that having friends who smoke and easy access to cigarettes from them encourages one to smoke [4]. One of the many reasons for smoking among medical students is to alleviate stress and anxiety, which increase severalfold during professional exams. Thus, exams influenced the smoking habits of 13.5% of students in this study and research conducted in Banu, Pakistan, which increased smoking in 69.8% of respondents [1]. Only 10% of students in this study admitted to smoking in the company of non-smokers, which is in contrast to an analysis performed in Prishtina, Kosovo, where the majority of students, i.e., 75%, admitted to it [15].

Cigarette smoking, its hazardous effects, and smoking cessation methods are integral to the medical curriculum. However, only 19.5% wanted to or tried to quit smoking in this study, and research conducted in Karachi, Pakistan, revealed that 15.13% wanted to and 17.93% tried leaving [6]. Another analysis performed in Taif, Saudi Arabia, showed contrasting results where 80% wanted to and 68.9% tried quitting smoking [3]. In this study, 5% of participants also sought professional guidance to quit smoking, slightly less than the results obtained in a study conducted in Karachi, Pakistan, where 11.8% of males and 6% of females sought advice from a doctor [20].

The prevalence of cigarette smoking in this study is an underestimation as the smoking status was disclosed by the participants themselves, which led to false reporting and subject bias. Additionally, smokers might have chosen not to participate despite ample reassurance that the confidentiality of data would be maintained, leading to self-selection bias. As the study was conducted at a single institute and all the respondents were medical students with higher levels of education, the results cannot be generalized to the entire population, thus adding to the limitations of this study. To counter this, equal representation was provided to each class and both genders to ensure maximum participation and a wide variety of perspectives.

Recommendations

Several proactive measures should be implemented to address the issue of smoking among college students. Firstly, smoking should be strictly prohibited within the college premises, including the campus and hostels. Rigorous monitoring and enforceable repercussions, such as fines, should accompany this prohibition in cases of rule violations. Secondly, it is essential to involve parents in the prevention process. Parents should be encouraged to maintain regular oversight of their children's activities and friendships to prevent the emergence of smoking habits among students. Thirdly, providing a range of extracurricular activities, including sports, is crucial. This allows students to channel their excess energy into productive and healthy pursuits, reducing the likelihood of turning to smoking. Fourthly, there is a need for specialized educational programs tailored for adolescents, with a particular focus on smoking cessation. These programs should be integrated into the curriculum for medical students, ensuring that future healthcare professionals are well-equipped to address smoking-related issues. Lastly, cost-effective interventions for smoking cessation should become a routine part of clinical care within the healthcare system. This will provide support and resources to students willing to quit smoking, making the process more accessible and practical. By implementing these measures, we can work towards a smoke-free college environment that promotes the health and well-being of all students.

## Conclusions

The study revealed that most participants had adequate knowledge and positive attitudes towards cigarette smoking, leading to a low frequency of tobacco consumption among them. However, certain habits that were found to be prevalent, like smoking in the company of non-smokers, need to be further addressed through appropriate educational tools and government legislation.
